# Clinical high risk for psychosis and service disengagement: Incidence and predictors across 2 years of follow‐up

**DOI:** 10.1111/eip.13599

**Published:** 2024-07-21

**Authors:** Fabio Catalano, Emanuela Leuci, Emanuela Quattrone, Derna Palmisano, Pietro Pellegrini, Simona Pupo, Marco Menchetti, Lorenzo Pelizza

**Affiliations:** ^1^ Department of Biomedical and Neuromotor Sciences Università di Bologna Bologna Italy; ^2^ Department of Mental Health Azienda USL di Parma Parma Italy; ^3^ Pain Therapy Service, Department of Medicine and Surgery Azienda Ospedaliero‐Universitaria di Parma Parma Italy

**Keywords:** clinical high risk, disengagement, early intervention in psychosis, outcome, ultra high risk

## Abstract

**Back:**

Service disengagement is common in subjects at CHR‐P (clinical high risk for psychosis), potentially worsening daily functioning and increasing the duration of untreated psychosis. That is why to identify baseline predictors of service disengagement could help better tailoring follow‐up on every CHR‐P individual.

**AIMS:**

Since there are few studies on this topic, the goals of this examination were: (1) to calculate service disengagement rates in a CHR‐P sample along 2‐years of follow‐up; and (2) to examine the most relevant predictive factors of disengagement at baseline.

**Methods:**

All young CHR‐P participants were enrolled within the ‘Parma At‐Risk Mental States’ (PARMS) protocol. At entry, the Global Assessment of Functioning (GAF) scale and the positive and negative syndrome scale (PANSS) were completed. Cox regression analyses were used.

**Results:**

Hundred and eighty CHR‐P subjects were recruited in this examination. During the follow‐up, a 2‐year service disengagement prevalence rate of 15% was observed. A statistically robust predictive factor of service disengagement was a lower prescription of antidepressant drug at entry. Other relevant baseline predictive factors were migrant status, higher GAF score, lower levels of anxious‐depressive symptoms and a lower acceptance of psychosocial interventions.

**Discussion:**

Baseline presence of anxious‐depressive features in CHR‐P individuals could favour engagement to specialized EIP services. However, implementing strategies to improve patients' motivation and involvement in care are needed.

## 1 INTRODUCTION

The first conceptualization of clinical high risk for psychosis (CHR‐P) mental states was made almost three decades ago (McGorry et al., 1995), following the stream of interest in psychosis prevention and the clinical relevance of DUP (duration of untreated psychosis) (Fusar‐Poli, 2017). Specifically, the CHR‐P status is defined by the presence of brief limited intermittent psychotic symptoms (BLIPS), attenuated positive symptoms (APS), or a combination of genetic risk indicators and recent functional decline (Ruhrmann et al., 2009).

Rates of psychosis conversion for people at CHR‐P range from 20% to 30% at 4 years (Fusar‐Poli et al., 2020), and there is plenty of evidence that CHR‐P subjects suffer from functional decline and high symptomatic burden (Fusar‐Poli et al., 2015). However, as shown for people with early psychosis, clinical management of CHR‐P individuals is complicated by an important percentage of service disengagement (Lal & Malla, 2015; Pelizza, Leuci, Quattrone, Azzali, Pupo, Paulillo, Pellegrini, & Menchetti, 2023a).

To our knowledge, there are only few studies on service disengagement in CHR‐P individuals and no other research conducted in Italy before. The first issue at this regard is that there is no universally accepted definition for mental healthcare engagement, neither for disengagement (Kreyenbuhl et al., 2009; O'Brien et al., 2009; Pelizza, Leuci, Quattrone, Azzali, Pupo, Paulillo, Pellegrini, & Menchetti, 2023b). Indeed, criteria employed for disengagement include referral failures from emergency services, missed initial appointments, non‐adherence with aftercare following hospitalization and other conceptualizations of intervention dropout (Green et al., 2011; Hengartner et al., 2017; Leanza et al., 2020). Clearly, the lack of consensus may have played a role in the large variation of reported prevalence rates across studies (McGorry, 2015).

Another interesting topic in this field is the possibility to detect predictors of service disengagement at baseline, as already examined for FEP population (Robson & Greenwood, 2022). The chance that DUP, substance use, symptom severity and insight at baseline could influence disengagement in CHR‐P population is attractive, but so far the few studies on this topic reported inconsistent results (Green et al., 2011; Hengartner et al., 2017; Leanza et al., 2020).

Therefore, the aims of this investigation were: (a) to examine the rate of service disengagement in a CHR‐P population recruited within an Italian early intervention service; and (b) to explore any relevant predictor of service disengagement at baseline as a tool for clinicians to identify the best strategy of management for each CHR‐P individual.

## 2 METHODS

### 2.1 Setting

Participants of the study were young people at CHR‐P referring to the ‘Parma At‐Risk Mental States’ (PARMS) program from 1st January 2016 to 31st December 2022. The PARMS is a diffuse service for ‘early intervention in psychosis’ (EIP) that has been implemented in all adult and adolescent mental healthcare centers of the Parma Department of Mental Health, in the Northern Italy (Pelizza, Leuci, Quattrone, Paulillo, & Pellegrini, 2023).

For the purposes of this research, inclusion criteria were: (a) specialist help‐seeking request; (b) age 12–35 years; (c) CHR‐P mental states as described in the ‘comprehensive assessment of at‐risk mental states’ (CAARMS) (Yung et al., 2005), authorized Italian version (Pelizza et al., 2019) (i.e., Brief Limited Intermittent Psychotic Symptoms [BLIPS], genetic vulnerability, and attenuated psychotic symptoms [APS]). Exclusion criteria of this investigation included: (a) previous non‐affective or affective psychotic episode in accordance with criteria of the Diagnostic and Statistical Manual of mental disorders, 5th Edition (DSM‐5) (APA, 2013); (b) neurological disease or any other somatic illness manifesting with psychiatric features; (c) known intellectual disability (i.e., Intelligence Quotient <70) and (d) previous exposure to Antipsychotics (AP) or current AP intake exceeding 4 weeks in the current episode of the disorder. In the present research, previous AP exposure was considered a proxy for a previous psychotic episode, consistently with the original version of the CAARMS criteria for psychosis threshold (Yung et al., 2005). Moreover, a current AP prescription of less than 4 weeks was required to minimize pharmacological interference with baseline psychopathological assessment (Pelizza et al., 2023d).

### 2.2 Instruments and assessment

For the specific goals of this research, clinical/sociodemographic information was retrospectively collected at entry by PARMS team members, based on both clinical interview and medical records (i.e., information on ethnic group, age, gender, migrant status, employment, years of education, civil and living status, previous hospitalization, past specialist contact, DUP, current substance abuse, previous suicide attempt, psychopharmacological prescriptions, family history of psychosis and acceptance of psychosocial interventions).

The psychopathological and functioning assessment included the global assessment of functioning (GAF) scale (APA, 2013), the positive and negative syndrome scale (PANSS) (Kay et al., 1987) and the Health of the Nation Outcome Scale (HoNOS) (Wing et al., 1998).

The PANSS is a clinical interview specifically assessing psychotic psychopathology, also in CHR‐P population (Pelizza, Leuci, Quattrone, Azzali, Paulillo, Pupo, Pellegrini, & Menchetti, 2023). As proposed by Shafer and Dazzi (2019), five main psychopathological factors were considered in this examination: negative symptoms, affect (depression/anxiety), disorganization, positive symptoms and resistance/excitement‐activity.

The GAF is a scale commonly used for the assessment of real‐world functioning in individuals with severe mental illness, including young people at CHR‐P (Catalan et al., 2022).

The HoNOS specifically assesses clinical and social outcomes in subjects with severe mental disorder, including early psychosis (Penno et al., 2017). As indicated by Wing et al. (1999), four main outcome domains were considered in this examination: psychiatric symptoms, social problems, impairment and behavioural problems.

### 2.3 Procedures

Following Robson and Greenwood (2022), we used the following definition of service disengagement: ‘a complete lack of contact or untraceable for at least 3 months despite a need of treatment, counted from the date of the last face‐to‐face meeting with the clinical staff’. This definition included CHR‐P subjects who actively contrasted further contact with the PARMS staff and were no longer traceable, and those who did not attend appointments for at least 3 months (Pelizza, Leuci, Quattrone, Azzali, Pupo, Paulillo, Menchetti, & Pellegrini, 2023). CHR‐P individuals meeting criteria for service disengagement (SD) were grouped in the CHR‐P/SD + (positive for service disengagement) subsample. The other subjects were included in the CHR‐P/SD− subgroup (negative for service disengagement).

As for condition of service disengagement in our CHR‐P total population, we calculated longitudinal incidence across the 2 years of follow‐up and any statistically significant association with clinical and sociodemographic characteristics at entry, as well as with specialized PARMS intervention components.

### 2.4 Statistical analysis

Collected data were examined using the Statistical Package for Social Science (SPSS) for Windows, version 15.0 (SPSS Inc., 2010). Statistical tests were 2‐tailed, with a significance *p* level set at 0.05. As for service disengagement, cumulative risk rates were calculated using the Kaplan–Meier survival analysis. Significant associations of service disengagement with sociodemographic, clinical and treatment features at entry were examined in the CHR‐P total group using Cox regression analysis. The statistically significant predictive factors were used as covariates within a multivariate Cox regression analysis to explore the strongest predictors for service disengagement. This two‐step statistical methodology was used to adapt the number of covariates to the size of our CHR‐P sample and the ratio of events per variable (EPV) (Sedgwick, 2013). Specifically, in multivariable models, an EPV which is too small may affect the accuracy (risk estimates) and precision (95% confidence intervals) of hazard ratios of the variables, resulting in misleading findings (van Domburg et al., 2014). Therefore, we selected a variable/participant ratio of 1:20 (i.e., 20 individuals for each covariate) and 10 EPV to maintain the validity of our statistical model (Riley et al.,  2019).

## 3 RESULTS

**FIGURE 1:**
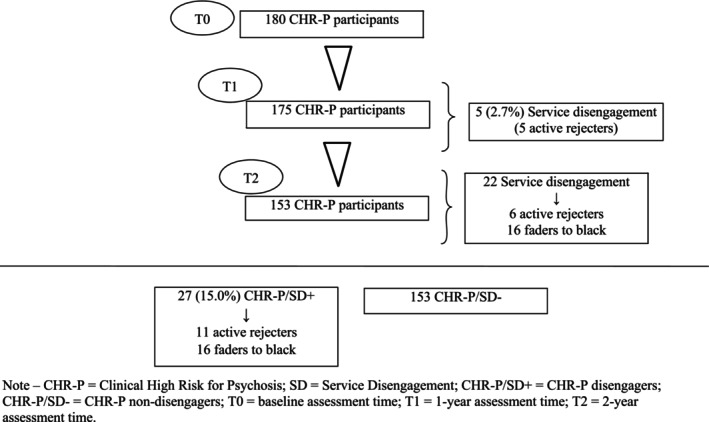
Service disengagement across the 2‐year follow‐up period in the CHR‐P total group (*n* = 180). Note—CHR‐P, clinical high risk for psychosis; CHR‐P/SD+, CHR‐P disengagers; CHR‐P/SD‐, CHR‐P non‐disengagers; SD, service disengagement; T0, baseline assessment time; T1, 1‐year assessment time; T2, 2‐year assessment time.

A total of 180 CHR‐P individuals were recruited in this examination. Five (2.7%) of them disengaged the PARMS program in the first year of treatment (Figure 1), actively refusing further contact with the PARMS team and being no longer traceable (‘active rejecters’). During the second year of follow‐up, other six active rejecters were lost and sixteen did not attend appointments for at least 3 months despite clinician's advice (‘faders to black’). This latter subgroup of ‘disengagers’ did not explicitly refuse treatment, but silently dropped out the PARMS protocol without being traceable anymore. The CHR‐P/SD+ subsample included 27 participants (15% of the total group: 11 active rejecters and 16 faders to black). The other 153 subjects were included in the CHR‐P/SD− subsample. The results of the Kaplan–Meyer survival analysis were shown in the Table 1.

**TABLE 1:** Kaplan–Meyer survival analysis results on service disengagement in the CHR‐P total sample (*n* = 180).

Variable	1‐cumulative proportion surviving at the time	
	Estimate	SE
1‐year service disengagement rate	.028	.012
2‐year service disengagement rate	.150	.027

Abbreviations: CHR‐P, clinical high risk for psychosis; SE, standard error.

As for service disengagement condition, univariate Cox proportional‐hazard models showed a statistically significant predictive role for migrant status, presence of BLIPS at baseline, lower PANSS ‘affect’ dimension score at entry, higher baseline GAF score, lower baseline HoNOS total score and HoNOS ‘psychiatric symptoms’ and ‘social problems’ domain subscores, lower baseline prevalence of antidepressant drug prescription, and lower baseline prevalence of individual psychotherapy and family psychoeducation acceptance (Table 2). However, the only parameter resulted statistically significant in the multivariate Cox proportional‐hazards model was represented by the lower baseline prevalence of antidepressant medication prescription (Table 3).

**TABLE 2:** Univariate Cox proportional‐hazard models for service disengagement condition in the two CHR‐P subgroups.

Variable	CHR‐P/SD+ (*n* = 27)	CHR‐P/SD‐ (*n* = 153)	Statistic test				
			B (SE)	HR	95% IC		*p*
					Lower Higher		
Gender (males)	13 (48.1%)	77 (50.3%)	−.044 (.385)	.957	.450	2.036	.909
Ethnic group (white Caucasian)	22 (81.5%)	137 (89.5%)	−.568 (.495)	.567	.215	1.496	.251
Migrant Status	8 (29.6%)	20 (13.1%)	.956 (.422)	.384	.168	.878	**.023**
Civil status (single)	21 (95.5%)	99 (98.0%)	−.644 (1.024)	.525	.071	3.093	.529
Living status (with parents)	18 (81.8%)	97 (96.0%)	.288 (1.225)	1.333	.121	4.704	.814
NEET	8 (36.4%)	29 (28.7%)	.874 (.341)	.533	.266	.976	.453
Age (at entry)	19.07 ± 3.65	19.61 ± 3.82	−.024 (.051)	.976	.883	1.079	.636
Education (in years)	10.67 ± 1.86	11.44 ± 2.52	−.117 (.082)	.890	.757	1.046	.156
DUI (in weeks)	35.85 ± 34.77	48.24 ± 50.74	−.006 (.005)	.994	.984	1.004	.233
Past hospitalization	3 (11.1%)	25 (16.3%)	−.342 (.612)	.710	.214	2.358	.576
Past specialist contact	13 (48.1%)	70 (45.8%)	.036 (.385)	1.037	.487	2.205	.926
Past attempted suicide	0 (0.0%)	19 (12.4%)	−3.175 (2.716)	.042	.001	8.850	.243
Family history of psychosis	6 (22.2%)	53 (34.6%)	−.549 (.463)	.577	.233	1.431	.235
Current substance abuse	4 (14.8%)	27 (17.6%)	−.209 (.542)	.812	.281	2.347	.700

CHR‐P subgroups							
APS	16 (59.3%)	123 (81.0%)	−.993 (.392)	.370	.172	.798	**.011**
BLIPS	8 (29.6%)	22 (14.4%)	.901 (.422)	2.463	1.078	5.628	**.033**
Genetic vulnerability	3 (11.1%)	8 (4.6%)	−.157 (.463)	.855	.345	2.118	.735
PANSS score							
Positive symptoms	10.50 ± 3.50	10.81 ± 3.84	−.019 (.059)	.981	.875	1.101	.749
Negative symptoms	17.18 ± 6.62	19.77 ± 7.81	−.040 (.030)	.961	.906	1.020	.193
Disorganization	14.27 ± 5.17	15.96 ± 5.36	−.056 (.046)	.945	.863	1.035	.224
Affect	13.18 ± 5.22	15.87 ± 5.08	−.087 (.043)	.916	.842	.998	**.045**
Resistance/excitement‐activity	8.05 ± 3.59	7.08 ± 3.12	.069 (.060)	1.072	.953	1.205	.249
Total score	65.41 ± 17.43	71.81 ± 17.25	−.019 (.013)	.981	.956	1.007	.155
GAF score	51.74 ± 9.56	48.20 ± 8.35	.044 (.023)	1.045	.999	1.092	**.049**

HoNOS score							
Behavioural problems	2.22 ± 2.14	2.75 ± 2.07	−.115 (.101)	.892	.731	1.088	.258
Impairment	1.96 ± 2.23	2.23 ± 1.98	−.064 (.105)	.938	.763	1.153	.544
Psychiatric symptoms	7.59 ± 3.56	8.95 ± 3.14	−.155 (.058)	.891	.796	.999	**.048**
Social problems	4.63 ± 2.70	6.46 ± 3.83	−.142 (.062)	.868	.769	.980	**.023**
Total score	16.41 ± 6.48	20.39 ± 8.02	−.067 (.028)	.935	.885	.989	**.019**
AP prescription	9 (33.3%)	83 (54.2%)	−.769 (.408)	.464	.208	1.032	.060
AD prescription	1 (3.7%)	42 (27.5%)	−2.132 (1.019)	.119	.016	.874	**.036**
MS prescription	1 (3.7%)	16 (10.5%)	−1.025 (1.019)	.359	.049	2.645	.315
BDZ prescription	4 (14.8%)	37 (24.2%)	−.560 (.542)	.571	.198	1.652	.301
Individual psychotherapy acceptance	5 (22.7%)	93 (60.8%)	−1.465 (.509)	.231	.085	.626	**.004**
Family psychoeducation acceptance	3 (13.6%)	60 (39.2%)	−1.270 (.621)	.281	.083	.949	**.041**
Case management acceptance	9 (40.9%)	91 (59.5%)	.655 (.343)	1.926	.823	4.506	.131

*Note*: Significant statistical p values are in bold.

Abbreviations: 95% CI = 95% confidence intervals for HR; AD, antidepressant drug; AP, antipsychotic drug; APS, attenuated psychotic symptoms; B, regression coefficient; BDZ, benzodiazepine drug; BLIPS, brief limited intermittent psychotic symptoms; CHR‐P, clinical high risk; CHR‐P/SD+, CHR‐P disengagers; CHR‐P/SD‐, CHR‐P non‐disengagers; DUI, duration of untreated illness; GAF, global assessment of unctioning; HR, hazard ratio; HoNOS, health of the nation outcome scale; MS, mood stabilizer drug; NEET, not in employment, in education, or in training; *p*, statistical significance; PANSS, positive and negative syndrome scale; SD, service disengagement; SE, standard error.

**TABLE 3:** Multivariate Cox proportional‐hazards models for service disengagement condition in the CHR‐P total sample (*n* = 180).

Service disengagement	B	SE	Wald	*p*	HR	95% CILower upper	
Migrant status	−.030	.615	.002	.961	.971	.291	3.240
BLIPS	.161	1.094	.022	.883	1.175	.138	10.023
PANSS ‘Affect’ factor score	−.031	.048	.427	.514	.969	.883	1.064
GAF score	.010	.031	.108	.742	1.010	.951	1.073
HoNOS ‘Psychiatric Symptoms’ domain score	−.034	.089	.146	.703	.967	.812	1.151
HoNOS ‘Social Problems’ domain score	.069	.089	.607	.436	1.071	.901	1.274
AP prescription	−.017	.512	.001	.974	.983	.361	2.682
AD prescription	−1.905	1.044	3.329	**.048**	6.717	.868	51.964
Individual psychotherapy acceptance	.815	.766	1.131	.287	2.259	.503	10.143
Family psychoeducation acceptance	.710	.932	.580	.446	2.034	.327	12.648
Overall (score) → *X* ^2^ = 23.809; df = 10; *p* = **.014**							

*Note*: Significant statistical *p* values are in bold.

Abbreviations: 95% CI, 95% confidence intervals for HR; AD, antidepressant drug; AP, antipsychotic drug; B, regression coefficient; CHR‐P, clinical high risk; df = degrees of freedom; GAF, global assessment of functioning; HoNOS, health of the nation outcome scale; HR, hazard ratio; *p* = statistical significance; PANSS, positive and negative syndrome scale; SE, standard error; *X* ^2^ = Chi‐square test value.

## 4 DISCUSSION

One of the main issues in the management of CHR‐P population is engagement to services (Salazar de Pablo et al., 2021), especially for its relevant association with poorer prognosis and greater mental healthcare costs across psychiatric services (O'Brien et al., 2009).

In this investigation, 15% of CHR‐P participants disengaged from the PARMS program during the 2 years of follow‐up. This finding is substantially in line with what (12% at 9 months) was reported in the OASIS (‘Outreach and Support in South London’) research (Green et al., 2011), but lower than those observed in the FePsy (‘Früherkennung von Psychosen’) and the NAPLS‐2 (‘North American Prodrome Longitudinal Study‐2’) studies, ranging between 35% at 2 years and 68% at 3 years (Hengartner et al., 2017; Leanza et al., 2020). However, although encouraging, our result may be partly related to a great heterogeneity across investigations, especially in terms of absence of a shared definition of service disengagement (Doyle et al., 2014) and difference in evaluation instruments and CHR‐P criteria used to define the study populations (Poletti et al., 2023).

Specifically, in this investigation we used a definition of service disengagement as ‘a complete lack of contact or no traceability for 3 months despite a need for treatment’ (considering the date of the last appointment) (Robson & Greenwood, 2022). Differently, Leanza et al. (2020) considered CHR‐P participants as disengaged when no appointment was established for 1 year after several attempts of clinical contact. Moreover, the same authors used the ‘basel screening instrument for psychosis’ (BSIP) to define CHR‐P mental states, also including a category of ‘unspecific risk’ for those subjects at lower risk because of manifesting less specific psychopathology and psychosis risk factors (Riecher‐Rössler et al., 2015).

In our CHR‐P population, the strongest baseline predictor for service disengagement was a lower baseline prescription of antidepressant medication. Together with lower severity levels of PANSS ‘affect’ dimension at entry, this finding suggests that the baseline presence of anxious‐depressive symptoms in CHR‐P individuals may favour engagement to specialized EIP services. In this respect, CHR‐P subjects frequently suffer from catastrophic fears relating to stigma, going mad, and distressing treatments (such as hospitalization), as well as from making sense of their unusual symptoms (Pelizza et al., 2022), thus being able to reactively develop anxiety and clinical depression (Pelizza, Leuci, Quattrone, Azzali, Paulillo, Pupo, & Pellegrini, 2023; Taylor et al., 2015). In this sense, depressive features could support patient's help‐seeking behaviour, acceptance of antidepressant prescription, and increased insight on symptoms, thus contrasting service disengagement in this young population.

The findings of our examination also found that lower acceptance of psychosocial interventions (especially individual psychotherapy and family psychoeducation) at baseline predicted service disengagement in CHR‐P individuals treated within the PARMS program. As investigated by Tranulis et al. (2011), these interventions could have beneficial effects on trust in EIP services and on medication adherence, which is considered a robust predictor correlated to leaving EIP programs in patients with first episode psychosis (FEP) (Fusar‐Poli et al., 2024).In this respect, treatment engagement should be always understood and planned as shared decision making among family members, patients and EIP staff (Alston et al., 2019). Moreover, our finding could also suggests a possible role of poorer intensive efforts by PARMS team professionals for those CHR‐P individuals poorly adherent at the program from the moment they entered in the therapeutic protocol and thus thought to be very likely to disengage. Therefore, implementing adequate strategies to enhance service engagement and care motivation, or to re‐engage CHR‐P individuals with poor treatment non‐adherence and low motivation to engage (also through text messaging, telehealth delivery and remote technologies) are needed (D'Arcey et al., 2020). In particular, interventions on patients for favouring decision making about their treatments and access to mental healthcare centers (e.g., individualized therapeutic‐rehabilitation plans shared together with family members and health professionals) (Pelizza et al., 2021), or/and treatments targeting cognition and motivation with the help of a motivational coach (Anderson et al., 2013) may also be useful.

Other additional predictors for service disengagement in our CHR‐P sample were related to lower baseline severity levels of psychopathology (i.e., lower HoNOS ‘psychiatric symptoms’ score, lower PANSS affect dimension subscore) and higher baseline socio‐occupational and daily functioning (i.e., higher GAF score and lower HoNOS ‘social problems’ domain subscore). In this respect, a reactive disengagement to individual circumstances (such as quick returning to school or work) can potentially exist, especially when supporting by attenuated severity of clinical picture and/or better self‐perceived performance and functioning. In this case, engagement with CHR‐P program may become a minor priority that individuals would choose if it does not impact on their main priority (i.e., the return to school/work) (Polillo et al., 2022). In line with this hypothesis, previous studies reported an improvement in negative symptoms before service disengagement of CHR‐P subjects, which was interpreted as inducing a reduced need for treatment and strictly related to a better social functioning (Hengartner et al., 2017; Leanza et al., 2020).

However, at the opposite side of what reported on less severe clinical picture at entry, also the baseline presence of BLIPS was another relevant predictive factor in our CHR‐population. Instead of favouring need for treatment and help‐seeking behaviour, overt psychotic symptoms seem to increase the risk of service disengagement. In this respect, the brief, limited and intermittent characteristics of these symptoms could induce the belief of their transitional nature, which once resolved spontaneously, no longer requires treatment and care. Alternatively, the presence of full‐blown psychotic features could be associated to poorer insight and higher treatment resistance, distancing CHR‐P individuals from EIP programs and mental healthcare services.

Finally, in line with the evidence reported in previous FEP research, our results also confirm the crucial role of migrant status as predictive factor of service disengagement. Indeed, minority groups are commonly less likely to consider the medical model of mental disorder, and accept mental health treatments less frequently (Maguire et al., 2020). Additionally, some ethnicities may experience the greatest stigma within their own communities, thus denying that their mental healthcare needs fit their cultural norms. The implementation of ethnic mediation services to encourage the engagement of migrant people in specialized mental health care programs is therefore fundamental in the EIP protocols (Tarricone et al., 2022).

### 4.1 Limitations

This study has noteworthy limitations. First, information on psychiatric antecedents was collected retrospectively, which means that the data was registered from medical records. This methodology is prone to information bias, as the data in the medical records may not be accurate or complete.

Another limitation was related to the absence of a general consensus on the definition of service disengagement. Indeed, this compromises comparisons across investigations and limits the generalization of our findings. We considered a coherent definition of engagement (Robson & Greenwood, 2022), but it is urgent to define and implement more cohesive methodologies in service disengagement research.

Moreover, our study did not consider the ‘true non‐engagers’ (i.e., CHR‐P subjects refusing any contact with the PARMS protocol from the start). In this sense, our sample may be considered as a sub‐population of help‐seeking individuals at CHR‐P. This probably biases the incidence of disengagement in the CHR‐P population usually seen in community, generalist mental healthcare centers. Indeed, CHR‐P subjects accepting to be recruited in the PARMS program could be more engaging and collaborative than those refusing EIP interventions from the start, thus impacting our findings.

As for statistical tests, a potentially critical problem regards the robustness of the multivariate Cox regression analysis for the sample size and the EPV number. Indeed, in multiple models, a ratio of 1:20 per covariate is not particularly strong, and an EPV which is too small can affect the accuracy and precision of hazard ratios. The consequence might be an incorrect significant association between the variable and outcome event (type I error), or an incorrect lack of association between a variable and the outcome event (type II error). On theoretical grounds, a variable/participant ratio of at least 1:20 and 10 EPV were suggested to maintain the validity of the model (Riley et al., 2019). However, future studies with larger CHR‐P samples and higher service disengagement incidence rates to confirm our interesting results are needed.

Lastly, our examination was limited to 2 years of follow‐up. Our findings are thus comparable with investigations having similar longitudinal designs. Further studies with longer duration are needed.

### 4.2 Conclusion

The findings of this examination showed that 15% of CHR‐P participants disengaged from our EIP protocol during 2 years of follow‐up. The strongest predictors were represented by a lower prescription of antidepressant drug at baseline. Together with lower baseline severity levels of affective features at entry, this findings suggests that anxious‐depressive symptoms may be considered as protective factors for prolonged engagement in specialized CHR‐P services. However, further investigations to clarify the relationship between clinical parameters and service disengagement are needed because of the uncertainty of evidence through the few studies on this topic.

## FUNDING INFORMATION

No specific grant from any funding agencies in the public, commercial or not‐for‐profit sectors aws received for this research. The PARMS service was partly financed through a treatment‐oriented regional fund (Progetto Esordi Psicotici della Regione Emilia‐Romagna) from January 2013 to December 2018.

## CONFLICT OF INTEREST STATEMENT

The authors declare no conflicts of interest.

## Data Availability

The data that support the findings of this study are available on request from the corresponding author. The data are not publicly available due to privacy or ethical restrictions.
